# Impact of cardiac rehabilitation on anxiety, depression, and health-related quality of life in cardiovascular patients

**DOI:** 10.1186/s43044-025-00658-8

**Published:** 2025-06-20

**Authors:** Estrella García-Sánchez, Mirian Santamaría-Peláez, Jerónimo J. González-Bernal, Josefa González-Santos, María Azucena Sedano García, Inmaculada De Juana Velasco, Jesús Sánchez Hernández, Héctor García Pardo, Jessica Fernández-Solana

**Affiliations:** 1 Hospital Universitario de Burgos, Burgos, Spain; 2 University of Burgos, Burgos, Spain; 3 Hospital Universitario Río Hortega, Valladolid, Spain

**Keywords:** Cardiac rehabilitation, Anxiety, Depression, Quality of life, Cardiovascular disease

## Abstract

**Introduction:**

The study aimed to evaluate the effects of a cardiac rehabilitation program based on physical exercise and the promotion of healthy habits on anxiety, depression, and health-related quality of life in patients with cardiovascular conditions. Additionally, it sought to analyze the influence of baseline anxiety and depression levels on post-treatment health-related quality of life outcomes.

**Methods:**

A longitudinal study was conducted with 189 patients who completed a structured cardiac rehabilitation program. Anxiety and depression were assessed using the Goldberg Anxiety and Depression Scale, while health-related quality of life was measured with the RAND-36 survey. Data were collected pre- and post-intervention. Statistical analyses included paired t tests for pre/post-comparisons and ANCOVA to evaluate the impact of initial anxiety and depression on health-related quality of life improvements.

**Results:**

The cardiac rehabilitation program significantly reduced anxiety (mean difference =  − 0.93, CI: − 1.42 to − 0.44, p < 0.001; Cohen’s d = 0.35) and depression (mean difference =  − 0.62, CI: − 0.99 to − 0.25, p < 0.001; Cohen’s d = 0.32), with improvements observed across several health-related quality of life dimensions, including emotional well-being (p = 0.005) and energy/fatigue (p < 0.001). Baseline anxiety and depression levels influenced changes in specific health-related quality of life dimensions, such as social functioning and role limitations due to physical health (p < 0.05). Causal interpretations are limited by the observational design and absence of a control group.

**Discussion:**

The results show an association between participation in cardiac rehabilitation programs and a reduction in anxiety and depression, as well as improved health-related quality of life in patients with cardiovascular disease. Baseline psychological status plays a key role in determining the magnitude of health-related quality of life improvements, highlighting the need for tailored interventions.

## Introduction

Cardiac rehabilitation programs (CRPs) are comprehensive, structured interventions designed to support the recovery of patients with cardiovascular disease, including those who have experienced myocardial infarction, heart failure, or undergone surgical procedures such as coronary artery bypass grafting. These programs, which integrate supervised physical exercise, health education, and psychological support, are a cornerstone secondary prevention and have demonstrated efficacy in reducing mortality, hospital readmissions, and improving both functional capacity and health-related quality of life (HRQoL) [1–7].

Despite these established benefits, psychological comorbidities—particularly anxiety and depression—remain highly prevalent in cardiac populations and are recognized as critical determinants of prognosis and quality of life [3, 8]. Studies report that the prevalence of depression among patients undergoing cardiac rehabilitation ranges from 15 to 45% following acute cardiac events, significantly higher than in the general population [3]. These psychological conditions are associated with poorer adherence to treatment, increased morbidity, and reduced HRQoL, underscoring the need for integrated mental health strategies within CRPs [9–11]. Despite robust evidence that CRPs improve both physical and psychological outcomes in patients with cardiovascular disease, significant gaps remain in our understanding of how baseline psychological status influences the effectiveness of these interventions. While systematic screening for anxiety and depression is recommended as a core component of cardiac rehabilitation, its implementation is inconsistent, and the impact of psychological comorbidities on rehabilitation outcomes is not fully understood [12, 13]. Furthermore, the long-term sustainability of psychological improvements and their relationship with HRQoL require further investigation [13, 14]. Thus, there is a need to evaluate not only the overall effect of a structured CRP on anxiety, depression, and HRQoL, but also by analyzing how baseline psychological state may modulate these outcomes, thus informing more personalized and effective rehabilitation strategies for cardiac patients [3, 10].

Improving psychological well-being is furthermore a fundamental target of CRPs, as mental health is closely linked to HRQoL and long-term outcomes [15]. Participation in CRPs has been shown to reduce symptoms of anxiety and depression and to enhance HRQoL, reinforcing the importance of a holistic approach that addresses both physical and psychological needs [16, 17]. Despite the recognized benefits, challenges persist in the implementation of CRPs, including low patient adherence and varying quality across programs [18]. However, challenges persist regarding patient adherence, variability in program quality, and disparities in access due to socioeconomic factors [8, 19].

A variety of psychological interventions have been incorporated into CRPs, including cognitive-behavioral therapy and mindfulness-based approaches, which have demonstrated short-term efficacy in alleviating anxiety and depression [17, 20]. Nevertheless, concerns remain about the sustainability of these effects and the identification of patient subgroups who benefit most from specific interventions. Additionally, the majority of studies have focused on short-term outcomes, with less attention given to the influence of baseline psychological status on the magnitude of benefit derived from CRPs.

HRQoL is a multidimensional construct that reflects patients’ perceptions of their physical, emotional, and social well-being. Its assessment is increasingly recognized as essential in evaluating the effectiveness of interventions for chronic diseases, including CVD [15]. Instruments such as the RAND-36 Health Survey provide valuable information on the impact of disease and treatment on patients’ daily lives and have been widely used in cardiac populations [21] (Orwelius et al. 2018).

Despite the robust evidence supporting CRPs, participation and adherence remain suboptimal, with only a fraction of eligible patients enrolling and completing these programs [8, 19]. Barriers such as motivation, perceived relevance, and socioeconomic disparities contribute to this gap, highlighting the need for tailored strategies to enhance engagement and optimize outcomes for diverse patient populations [16] (Bush et al. 2023).

In this context, the present study aims to evaluate the effect of a structured cardiac rehabilitation program based on physical exercise and the promotion of healthy habits on anxiety, depression, and health-related quality of life in patients with cardiovascular disease. Additionally, it seeks to determine whether baseline levels of anxiety and depression influence improvements in HRQoL following rehabilitation. By addressing these questions, this research contributes to a better understanding of the psychological benefits of CRPs and the factors that may optimize patient outcomes.

Efforts have been made to enhance patient engagement and accessibility within cardiac rehabilitation services. For instance, providing psychological assessments within the context of cardiac outpatient clinics reduces stigma and promotes attendance. Programs have also demonstrated improved patient acceptance of psychological referrals compared to traditional mental health services, highlighting the importance of integrated care in addressing the comprehensive needs of patients [3, 8].

## Methods

### Participants

A total of 189 patients with cardiac pathology were included in the study. The most frequent cardiac diagnosis among participants was ischemic heart disease, present in 182 patients (96.3%). Other conditions included arrhythmias (n = 26, 13.8%), valvular heart disease requiring intervention (n = 9, 4.8%), and heart failure (n = 6, 3.2%). Additionally, 11 patients (5.8%) had implanted cardiac devices such as pacemakers or defibrillators. After being referred by the cardiologist in charge of the Cardiac Rehabilitation Unit for assessment of their suitability for participation in the outpatient physical training component of the CRP, these patients subsequently completed the program.

The inclusion criteria for the study were as follows: (i) confirmed diagnosis of cardiovascular pathology; (ii) attendance at the Cardiac Rehabilitation Unit consultation following referral from the Cardiology department; (iii) provision of written informed consent specific to this study; and (iv) completion of the Cardiac Rehabilitation Program (CRP). Exclusion criteria comprised: (i) inability to participate in the physical exercise program due to medical reasons; (ii) refusal to participate in the program; and (iii) failure to complete the CRP. This study was approved by the Ethical Committee for Research with Medicines of the Health Area of Burgos and Soria (Ref. CEIm 2569) on June 22, 2021. The research was conducted in accordance with the guidelines set out in the World Medical Association’s Declaration of Helsinki. Data collection was carried out in participating centers by designated personnel, and the data were anonymized before sharing with the research team, remaining anonymous and aggregated from that point forward.

### Procedure

A longitudinal cohort study was conducted with a single experimental group composed of patients with cardiovascular pathology. No sample size calculation was performed, and a control group was not included in this study due to ethical considerations. Although no formal priori power analysis was performed, the study included all eligible patients referred to the cardiac rehabilitation program during the study period. The final sample of 189 participants provided sufficient statistical power to detect significant changes in the primary outcomes, as supported by the effect sizes and confidence intervals reported. Given that the treatment in question is aimed at improving the well-being of participants and has the potential to provide direct benefits to their health, it was considered inappropriate to deprive any group of access to this treatment. Instead of a traditional control group, an intervention design was chosen to ensure that all participants receive the treatment, thereby ensuring equity and benefit for all everyone involved in the study.

**Fig. 1:**
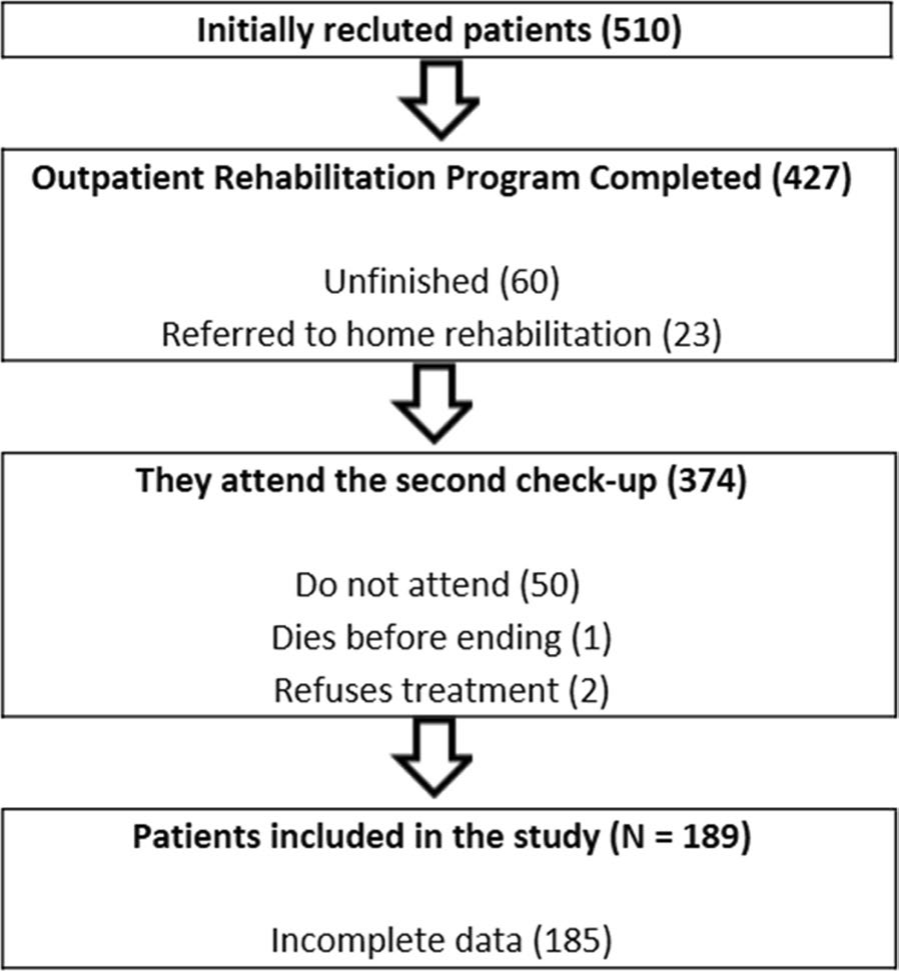
Flowchart

After confirming that the inclusion criteria were met, data were collected from each patient during their initial consultation at the Rehabilitation Service of the University Hospital of Burgos. This pretest assessment included individuals who were considered likely candidates for participation in a CRP. Data collection was performed by a physician specializing in physical medicine and rehabilitation. Clinical data were extracted from patients’ medical records, while sociodemographic information and assessment scale scores were obtained during the consultation. Figure 1 shows the flowchart for this research.

Following referral by their cardiologists, the patients were admitted to the Cardiac Rehabilitation Unit of the University Hospital of Burgos. At the initial consultation, a cardiologist obtained a detailed clinical history, including anamnesis and evaluation of complementary tests such as lipid profile analysis, electrocardiogram and echocardiogram. In the absence of a recent stress test, either ergometry or cardiopulmonary exercise testing was performed. Subsequently, in the nursing consultation, cardiovascular risk factors, dietary habits, smoking history, and social environment were assessed, with health education and documentation explaining the program distributed. Afterward, the rehabilitation physician reviewed clinical history, confirmed adherence to medical treatment, and performed a general physical examination to determine each patient’s suitability for participating in the CRP. Finally, patients were interviewed by a physiotherapist from the Cardiac Rehabilitation Unit, who again explained the CRP and supervised its implementation.

The variables collected included biological gender, distance from the place of residence to the rehabilitation center, age, anxiety, depression, and HRQoL. Some variables were self-reported by the patient, while others objectively measured during the consultation.

The CRP implemented in this study was designed to comprehensively address the needs of patients with cardiovascular pathologies, incorporating supervised physical exercise, psychological support and health education as core components.

The CRP was conducted over a period of 1–2 months, with training sessions scheduled two to three times per week. Each session was structured into four phases:

1. Phase 1: Warm-up (10 min): Mobilization of large muscle groups through flexion, extension, lateralization, and rotation exercises targeting the cervical region, upper limbs, trunk, and lower limbs. Each exercise was performed approximately five times.
2. Phase 2: Aerobic Exercise (35 min): Conducted on cyclo-ergometers or treadmills, with intensity adjusted according to the maximum heart rate frequency (HRF) obtained in the stress test, using the Karvonen formula [22, 23]. During the first third of the sessions, exercise was performed at 50% of the MHRF, increasing to 60% in the second third, and reaching 80% in the final third [24–26]. In patients evaluated by ergo spirometry, the intensity was established to achieve the heart rate obtained between the first and second ventilatory threshold [24, 27]. In general, continuous training was initiated, seeking an objective heart rate at a sustained exercise intensity. Subsequently, the patient was switched to an interval regime based on the results of the Conconi test [28].
3. Phase 3: Strength Training (twice per week): Focused on large muscle groups, utilizing 1–2 kg weights and performing four to five repetitions per exercise. Emphasis was placed on proper technique and controlled breathing, with exhalation during exertion and inhalation during relaxation.
4. Phase 4: Stretching and Flexibility (5–10 min): Involved static stretching and balance exercises to improve flexibility and facilitate the return to resting state.

Recognizing the importance of psychological well-being in cardiac rehabilitation, the program incorporated group or individual psychological therapy sessions, tailored to each patient’s needs and delivered by a clinical psychologist once every two weeks. In addition, relaxation workshops led by a physiotherapist were provided to all participants, lasting 20–30 min and focusing on diaphragmatic breathing techniques and progressive relaxation.

Furthermore, weekly one-hour educational sessions were conducted, covering topics such as heart-healthy nutrition, understanding cardiovascular disease, relevant pharmacology, physical activity and sexual health, cardiovascular risk factors, and smoking cessation strategies.

Before each session, vital parameters such as blood pressure and heart rate were recorded. Abdominal circumference and body weight were measured on a weekly basis. Patients requiring additional support were referred to the Smoking Unit, Clinical Psychology or Mental Health, as appropriate.

Three months after completion of the CRP (posttest), a follow-up data collection was conducted. The data were then entered into a database for subsequent statistical analysis. During the same consultation, the importance of maintaining the lifestyle changes achieved during the program was emphasized.

### Instruments

HRQoL is measured with the 36-item RAND Health Survey (RAND-36), a generic instrument applicable to a wide range of health conditions and populations [29, 30]. Its reliability and applicability in cardiovascular populations have been confirmed in recent comparative studies of SF-36 and RAND-36 [21]. For each dimension, item scores are first recoded and then averaged to generate a domain score. The eight dimensions assessed are: physical functioning (PF), role limitations due to physical health (RP), pain (BP), general health (GH), energy/fatigue (VT), social functioning (SF), role limitations due to emotional problems (RE), and emotional well-being (MH). Additionally, item 2 assesses health transition (HT) and is scored separately [29].

Anxiety and depression are measured with the Goldberg Anxiety and Depression Scale (GADS). The GADS is a short and simple questionnaire that includes two distinct subscales, one for anxiety and one for depression [31]. In the anxiety subscale, four questions are asked initially, and if the patient responds affirmatively to two or more, five additional questions are asked. The cutoff point for identifying possible significant anxiety is 4 or more points on the total scale. In the depression subscale, if the patient responds affirmatively to at least one of the initial questions, the rest of the questionnaire is continued, and a score of 2 or more indicates possible significant depression [32].

The scale has been shown to have a sensitivity of 83.1% and a specificity of 81.8%, which makes it suitable for correctly identifying those patients with psychiatric morbidity and ruling out those without relevant disorders [32] making it an effective screening tool. In addition, the EADG allows estimating the severity of the pathology, as higher scores correlate with higher levels of impairment [31].

In this research, total anxiety and depression scores are used as quantitative variables, but cutoff points that establish dichotomous categorical variables (yes/no anxiety or depression) are also used.

### Statistical analysis

The IBM SPSS Statistics software version 25.0 was used for data analysis.

First, a descriptive analysis of the variables was carried out. Quantitative variables are described with the mean and standard deviation and qualitative variables with the distribution of frequencies and percentages. The normality of the dataset was assessed using the Kolmogorov–Smirnov test.

To check whether the CRP based on physical exercise and promotion of healthy habits improves anxiety, depression, and HRQoL in patients with cardiac pathology, Student’s t statistical analysis was performed for related samples. In order to check whether the presence of anxiety and/or depression at the beginning of the rehabilitation treatment influences the HRQoL achieved after said treatment, the ANCOVA statistic was used in which the dependent variable was the differential HRQoL score (obtained from the T2-T1 transformed scores for each dimension of the RAND-36 scale following its scoring rules), the covariate was the pretest score of the HRQoL dimensions in each case and the fixed factor was the presence or absence of anxiety or depression. No correction for multiple comparisons was applied, as each analysis compared only two independent groups.

## Results

The sample consisted of 189 patients, 157 (83.1%) were male, and 32 (16.9%) were female; 145 (76.7%) reside in the locality where the rehabilitation program takes place, 23 (12.2%) patients at a distance of less than 60 km from this locality, and 21 (11.2%) at a distance of more than 60 km. The mean age of the participants was 60.68 years with a maximum of 84 and a minimum of 15.

Table 1 summarizes the sociodemographic and clinical characteristics of participants at baseline.

**Table 1:** Baseline characteristics table

Variable	Value
Age (mean ± SD)	60.68 ± 10.89
Biological gender, n (%)	
Male	157 (83.1%)
Female	32 (16.9%)
Place of residence, n (%)	
Burgos	145 (76.7%)
< 60 km	23 (12.2%)
> 60 km	21 (11.1%)
Ischemic heart disease	182 (96.3%)
Heart failure	6 (3.2%)
Valvular disease (surgically treated)	9 (4.8%)
Arrhythmias	26 (13.8%)
Implanted cardiac device	11 (5.8%)
Hypertension	98 (51.9%)
Dyslipidemia	106 (56.1%)
Diabetes Mellitus	54 (28.6%)
Anxiety (mean ± SD)	2.89 ± 3.13
Depression (mean ± SD)	1.89 ± 2.27
Anxiety (GADS ≥ 4), n (%)	51 of 142 (35.9%)
Depression (GADS ≥ 2), n (%)	64 of 142 (45.1%)

Values are presented as mean ± standard deviation (SD) for continuous variables and as number (percentage) for categorical variables. GADS = Goldberg Anxiety and Depression Scale

The descriptive statistics of the quantitative variables such as anxiety, depression, and quality of life in the pretest and posttest are shown in Table 2.

**Table 2:** Descriptive statistics of anxiety, depression, and health-related quality of life

	Minimum	Maximum	Mean	SD
Anxiety pretest	0	9	2.89	3.13
Anxiety posttest	0	9	1.91	2.77
Depression pretest	0	9	1.89	2.27
Depression posttest	0	8	1.02	1.81
RAND_PF pretest	4.00	37.00	19.28	6.44
RAND_PF posttest	6.00	42.00	18.38	6.78
RAND_RP pretest	0.00	100.00	29.54	37.03
RAND_RP posttest	0.00	100.00	46.11	42.21
RAND_BP pretest	0.00	100.00	68.85	24.23
RAND_BP posttest	0.00	100.00	71.92	25.79
RAND_GH pretest	5.00	90.00	53.50	17.25
RAND_GH posttest	5.00	100.00	58.17	19.08
RAND_VT pretest	10.00	90.00	57.98	16.18
RAND_VT posttest	10.00	90.00	63.83	16.10
RAND_SF pretest	0.00	100.00	77.48	23.60
RAND_SF posttest	0.00	100.00	84.09	21.60
RAND_RE pretest	0.00	100.00	55.88	44.92
RAND_RE posttest	0.00	100.00	63.45	42.92
RAND_MH pretest	16.00	88.00	64.00	16.41
RAND_MH posttest	20.00	88.00	66.63	15.69
RAND_2 pretest	0.00	100.00	40.49	23.48
RAND_2 posttest	0.00	100.00	67.57	26.88

SD: standard deviation. RAND: RAND 36-Item Health Survey; PF: Physical Functioning; RP: Role limitations due to physical health; BP: Pain; GH: General Health; VT: Energy/Fatigue; SF: Social Functioning; RE: Role limitations due to emotional problems; MH: Emotional Well-being; and RAND_2: Reported health transition item

The results of the pre- and post-intervention comparisons for the variables assessed, including anxiety, depression, and HRQoL dimensions, are presented below (Table 3). While most health-related quality of life dimensions showed improvements following the intervention, the physical functioning domain (RAND_PF) was the only one that demonstrated a slight decrease in mean score. To complement the interpretation of statistical significance, effect sizes (Cohen’s d) were calculated and included, providing information on the magnitude and clinical relevance of the observed changes.

**Table 3:** T test results for paired samples

Variable	Mean difference (95% CI)	t-value	p value	Cohen’s d
Anxiety	0.93 (0.44, 1.42)	3.76	**<** **0.001**	0.35
Depression	0.62 (0.26, 0.99)	3.42	**<** **0.001**	0.29
RAND_PF	0.90 (0.11, − 1.91)	1.75	**0.041**	0.13
RAND_RP	− 16.57 (− 22.79, − 10.34)	− 5.25	**<** **0.001**	0.39
RAND_BP	− 3.07 (− 6.67, 0.54)	− 1.68	**0.048**	0.12
RAND_GH	− 4.67 (− 6.81, − 2.54)	− 4.32	**<** **0.001**	0.33
RAND_VT	− 5.85 (− 7.94, − 3.77)	− 5.54	**<** **0.001**	0.43
RAND_SF	− 6.60 (− 10.00, − 3.19)	− 3.82	**<** **0.001**	0.29
RAND_RE	− 7.57 (− 14.29, − 0.86)	− 2.22	**0.014**	0.17
RAND_MH	− 2.63 (− 4.60, − 0.65)	− 2.63	**0.005**	0.20
RAND_2	− 27.08 (− 31.34, − 22.82)	− 12.54	**<** **0.001**	0.94

CI: Confidence Interval; RAND: RAND 36-Item Health Survey; PF: Physical Functioning; RP: Role limitations due to physical health; BP: Pain; GH: General Health; VT: Energy/Fatigue; SF: Social Functioning; RE: Role limitations due to emotional problems; MH: Emotional Well-being; and RAND_2: Reported health transition item. Values in bold indicate statistically significant results (*p* < 0.05)

Table 4 shows that the presence of anxiety at the beginning of the rehabilitation program influences the improvement in the RP, BP, and MH dimensions of HRQoL achieved after treatment.

**Table 4:** ANCOVA analysis between anxiety at the beginning and non-anxiety at the beginning groups related to HRQoL improvements

Variables	Treatment group	Mean difference	SD	P	Observed power
RAND_PF	Anxiety	− 1.11	7.42	0.902	0.000
	Non-anxiety	0.17	7.29		
RAND_RP	Anxiety	12.94	42.14	**0.047**	0.028
	Non-anxiety	17.14	44.77		
RAND_BP	Anxiety	− 2.36	28.76	**0.035**	0.032
	Non-anxiety	2.30	21.56		
RAND_GH	Anxiety	4.62	15.94	0.466	0.004
	Non-anxiety	3.71	12.59		
RAND_VT	Anxiety	6.73	16.66	0.121	0.017
	Non-anxiety	5.14	13.96		
RAND_SF	Anxiety	11.11	24.73	0.130	0.016
	Non-anxiety	3.24	20.29		
RAND_RE	Anxiety	18.88	42.84	0.109	0.018
	Non-anxiety	2.41	45.78		
RAND_MH	Anxiety	3.39	15.61	**0.004**	0.059
	Non-anxiety	1.74	12.49		
RAND_2	Anxiety	26.39	28.17	0.459	0.004
	Non-anxiety	24.50	31.00		

SD: Standard Deviation; RAND: RAND 36-Item Health Survey; PF: Physical Functioning; RP: Role limitations due to physical health; BP: Pain; GH: General Health; VT: Energy/Fatigue; SF: Social Functioning; RE: Role limitations due to emotional problems; MH: Emotional Well-being; and RAND_2: Reported health transition item. Values in bold indicate statistically significant results (*p* < 0.05)

Table 5 shows that the presence of depression at the beginning of the rehabilitation program influences the improvement in the SF and MH dimensions of HRQoL achieved after treatment.

**Table 5:** ANCOVA analysis between depression at the beginning and non-depression at the beginning groups related to HRQoL improvements

Variables	Treatment group	Mean difference	SD	P	Observed power
RAND_PF	Depression	− 1.09	7.12	0.921	0.000
	Non-depression	0.37	7.49		
RAND_RP	Depression	21.18	39.40	0.699	0.001
	Non-depression	11.08	46.76		
RAND_BP	Depression	3.48	28.07	0.923	0.000
	Non-depression	−1.70	20.81		
RAND_GH	Depression	5.82	15.66	0.935	0.000
	Non-depression	2.57	12.05		
RAND_VT	Depression	8.16	17.13	0.265	0.009
	Non-depression	3.71	12.65		
RAND_SF	Depression	7.86	26.06	**0.011**	0.046
	Non-depression	4.60	18.54		
RAND_RE	Depression	14.36	43.02	0.062	0.025
	Non-depression	3.37	46.77		
RAND_MH	Depression	3.73	15.67	**0.024**	0.036
	Non-depression	1.19	11.73		
RAND_2	Depression	30.48	29.20	0.597	0.002
	Non-depression	20.18	29.99		

SD: Standard Deviation; RAND: RAND 36-Item Health Survey; PF: Physical Functioning; RP: Role limitations due to physical health; BP: Pain; GH: General Health; VT: Energy/Fatigue; SF: Social Functioning; RE: Role limitations due to emotional problems; MH: Emotional Well-being; and RAND_2: Reported health transition item. Values in bold indicate statistically significant results (*p* < 0.05)

## Discussion

The present study aimed to evaluate the effects of a structured cardiac rehabilitation program based on physical exercise and the promotion of healthy habits on anxiety, depression, and health-related quality of life in patients with cardiovascular disease. Additionally, it sought to determine whether baseline levels of anxiety and depression influenced the improvements in HRQoL following rehabilitation.

Evidence highlights a strong correlation between psychological health and cardiovascular disease risk. Chronic stress and negative mental states, such as anxiety and depression, are linked to the accelerated development of cardiovascular risk factors, which can lead to adverse health outcomes [9, 11, 33]. In line with these findings, our study showed a significant association between the reduction of anxiety and depression following CRPs, highlighting the fundamentals of addressing psychological health in cardiac rehabilitation. These improvements align with the American Heart Association’s emphasis on the necessity for integrated approaches that target both psychological and physical well-being in cardiac care [33]. Some of the studies cited involve targeted psychological therapies, such as CBT or mindfulness-based interventions. While these differ from the structure of the cardiac rehabilitation program evaluated here, they support the broader evidence that addressing psychological health contributes to improved outcomes in cardiac populations. Furthermore, our results highlight the importance of tailoring interventions to address baseline psychological conditions, as patients with higher initial levels of anxiety and depression showed differential improvements in HRQoL dimensions, as shown in the results of this study. Promoting awareness and proactive management of mental health, as evidenced by the outcomes in this study, is essential for enhancing the overall recovery and quality of life of cardiac patients.

As mentioned above, anxiety disorders are prevalent among patients with coronary heart disease and have been linked to poorer clinical outcomes, including increased mortality and new cardiac events [11]. In line with this evidence, our study found that participation in a CRPs significantly reduced anxiety levels among patients, highlighting the relationship of exercise-based rehabilitation programs in alleviating psychological distress. These findings are consistent with a meta-analysis demonstrating that exercise therapy can significantly relieve anxiety symptoms in cardiac patients [9, 33].

On the other hand, in line with the second objective of the study, a comparison of baseline characteristics between patients with and without depressive tendencies revealed that 72.5% of those who were depressed at the 9-month mark had also shown depressive symptoms at baseline, compared to only 40.9% of the non-depressed group [10]. Similarly, our study found that baseline depression significantly influenced improvements in HRQoL dimensions, such as SF and MH, underscoring the lasting impact of initial depressive symptoms on rehabilitation outcomes. Additionally, patients with depressive tendencies were more likely to utilize maladaptive coping strategies, such as “abandonment or resignation,” which is associated with a higher likelihood of ongoing depressive symptoms [10]. Conversely, those who engaged in adaptive coping strategies, like “planning” and “positive interpretation,” were less likely to exhibit depressive tendencies at follow-up [10]. These findings align with our results, emphasizing the need for psychological support within CRPs to foster adaptive coping strategies and optimize recovery trajectories, particularly for patients with high baseline depression.

Efforts to improve patient participation and accessibility in cardiac rehabilitation have included integrating psychological assessments into outpatient cardiac clinics, which helps reduce stigma and promotes attendance. Such integrated care models have led to greater patient acceptance of psychological referrals compared to traditional mental health services, underscoring the importance of addressing the comprehensive needs of cardiac patients [3, 8]. Substantial evidence indicates that participation in CRPs results in significant improvements in HRQoL, as observed in our study, which found notable gains in domains such as RP, SF, and MH. Additionally, previous research has shown that post-rehabilitation physical activity can reduce cardiovascular mortality by up to 35%, improve functional capacity, and increase rates of return to work [15]. These findings highlight the dual benefit of CRPs in addressing both physical and psychological health, as improvements in HRQoL were accompanied by reductions in anxiety and depression. For patients with advanced heart failure or severe cardiac conditions, prioritizing HRQoL as a key treatment outcome is essential, as it provides valuable insight into the patient’s subjective experience—including psychological aspects like anxiety and depression—that are commonly associated with cardiac disease [15, 34].

In general, research in this field, including the present study, faces several limitations. Gender disparities in representation may lead to bias or limit the generalizability of results, as it is known that men and women experience different psychological and physical responses to cardiac rehabilitation. The diverse methodologies employed to measure psychological outcomes, such as the use of different psychometric scales, add complexity when comparing results across studies. Additionally, the reliance on self-reported measures introduces response bias, where participants may overstate improvements due to social desirability or other subjective factors. Although this bias is moderate, it highlights the need for complementary objective measures in future research. Furthermore, inconsistencies in intervention duration and follow-up periods may attenuate observed effects, complicating the analysis of cardiac rehabilitation interventions [16, 17]. Moreover, subgroup analyses by specific cardiac conditions were not feasible due to the overwhelmingly high proportion of participants with ischemic heart disease. Similarly, age-related subgroup analyses were not conducted, as the vast majority of participants were older adults, and the inclusion of younger patients was minimal. These factors limit the ability to generalize the effects of the intervention to other cardiac diagnoses or to populations with different age profiles. Finally, the absence of a control group limits the ability to definitively attribute observed improvements in anxiety, depression, and health-related quality of life solely to the cardiac rehabilitation program. This may inflate the perceived impact, and although difficult to quantify, the magnitude of this effect could be significant.

Despite these limitations, this study has notable strengths. It provides robust evidence of significant improvements in anxiety, depression, and HRQoL following participation in a CRP, emphasizing the importance of integrating psychological support into rehabilitation protocols. The longitudinal design allowed for the assessment of changes over time, adding depth to the analysis. Moreover, the use of validated instruments ensures reliability and facilitates comparability with previous research.

Future research should incorporate comparison groups that would allow for more robust causal inferences regarding the intervention’s effectiveness. Additionally, ensuring a more diverse sample, including a balanced representation of genders, would enhance the generalizability of the findings. Studies should also consider adjusting for potential confounders, such as age, comorbidities, and socioeconomic status, to better understand the factors influencing outcomes.

## Conclusions

This study shows that CRPs based on physical exercise and the promotion of healthy habits are associated with a significant reduction in anxiety and depression, while also improving HRQoL in patients with cardiovascular disease. The findings underscore the importance of addressing psychological factors, since the presence of anxiety and depression at the beginning of the program significantly influences the magnitude of improvements achieved in key HRQoL dimensions, such as emotional well-being, social functioning, and role limitations due to physical problems.

These findings underscore the need to implement integrated and personalized interventions that address both the physical and psychological aspects of the recovery process. Systematic assessment of patients’ psychological status at the beginning of rehabilitation would allow for the identification of those at higher risk of poorer outcomes and enable the adaptation of therapeutic strategies to maximize benefits.

## Data Availability

The datasets presented in this article are not readily available because the data are part of an ongoing study. Requests to access the datasets should be directed to mspelaez@ubu.es.
